# Assessing biomedical research capacities in selected countries of Latin America: challenges, opportunities, and recommendations

**DOI:** 10.3389/frma.2025.1594303

**Published:** 2025-07-04

**Authors:** Jorge A. Huete-Perez, Narayana Salvatierra

**Affiliations:** ^1^Science, Technology and International Affairs, Georgetown University, Washington, DC, United States; ^2^Center for Community, Service and Justice, Loyola University Maryland, Baltimore, MD, United States

**Keywords:** biomedical research, Latin America, research capacity, science policy, R&D investment, global partnerships

## Abstract

Despite increasing scientific output, biomedical research in Latin America remains unevenly developed, particularly in countries that are often overlooked in regional science policy discussions. This study assesses research capacities in Colombia, Costa Rica, Guatemala, Panama, and Peru, identifying key challenges, opportunities, and strategies to strengthen the region's scientific landscape. Using a mixed-methods approach—including surveys, expert interviews, and data analysis—this study examines infrastructure, institutional support, funding mechanisms, researcher training, and international partnerships. Additionally, it evaluates the impact of global programs, such as the Pew Latin American Fellows Program, in advancing research capacity. Findings highlight substantial differences in national R&D investment, workforce development, and institutional capabilities. Colombia and Costa Rica exhibit more developed research ecosystems, while Guatemala, Panama, and Peru face constraints such as limited national funding, dependency on external grants, and gaps in PhD/postdoctoral training. However, emerging opportunities include specialization in key biomedical fields, notably infectious diseases, genomics, and biotechnology, strengthening global partnerships, and leveraging research networks to address Latin America's pressing health challenges. This study contributes to ongoing discussions on regional science policy and international collaboration by addressing knowledge gaps and providing evidence-based recommendations for research funding, institutional development, and workforce expansion. To foster long-term growth, it recommends increasing national R&D investment, modernizing research infrastructure, expanding doctoral and postdoctoral training, and strengthening institutional and global research partnerships. By implementing targeted policies and institutional strategies, Latin America can enhance its role in global biomedical research and innovation while addressing regional health priorities.

## Introduction

### Background and rationale

Scientific research and technological innovation are recognized globally as essential drivers of progress and sustainable development (Messerli et al., 2019). In Latin America, scientific output has grown significantly, with indexed publications increasing by nearly 29% between 2015 and 2019 (RICYT, 2020). However, research capacity across the region remains uneven. While Brazil, Mexico, and Argentina lead in scientific productivity (Albornoz, 2002; Leta et al., 2021), many other countries face persistent challenges such as underinvestment, inadequate infrastructure, and limited human resources. These barriers hinder the development of robust research ecosystems.

Biomedical research is particularly critical for addressing global health challenges, fostering innovation, and addressing public health challenges (Ong, 2023). Recent advances in fields such as AI-driven autonomous experimentation systems have accelerated progress in drug discovery, nanomedicine, and precision oncology (da Silva, 2024). For Latin America, strengthening biomedical research capacities is not only vital for healthcare improvements but also for enhancing regional competitiveness in science. Effective research systems enable countries to tackle diseases relevant to their epidemiological landscapes while contributing to global scientific advancements.

Despite its importance, biomedical research in many Latin American countries is constrained by low R&D investment (ECLAC, 2022), insufficient integration into global scientific networks, and limited educational opportunities. These challenges disproportionately affect less-developed nations in the region, which often lack the resources to train scientists or build sustainable research infrastructures. Furthermore, the academic focus on more scientifically advanced countries leaves critical gaps in understanding the unique barriers faced by smaller or less-resourced nations. Addressing these disparities is essential for ensuring equitable participation in scientific innovation and for tailoring solutions to regional health needs.

### Objectives of the study

This study assesses biomedical research capacities in selected Latin American countries to identify key challenges and opportunities. It aims to provide actionable recommendations for strengthening research ecosystems and fostering international collaborations. Specifically, the study evaluates infrastructure and institutional support systems, examines obstacles in training and workforce development, and analyzes the role of international programs, such as the Pew Latin American Fellows Program, in building capacity. By focusing on Colombia, Costa Rica, Guatemala, Panama, and Peru—countries with diverse socioeconomic conditions but untapped potential—the study offers insights into barriers limiting research growth and strategies to enhance funding mechanisms.

### Significance of the study

Persistent gaps in funding, infrastructure, and training underscore the need for targeted strategies to support biomedical research in Latin America. This study contributes to ongoing discussions on regional science policy and international cooperation by addressing knowledge gaps and offering evidence-based recommendations. The findings aim to guide policymakers, funding agencies, and academic institutions in designing interventions that strengthen biomedical research ecosystems. Additionally, the study highlights the importance of international programs in fostering collaboration and advancing regional scientific capacity.

## Methodology

### Study design overview

**Figure 1:**
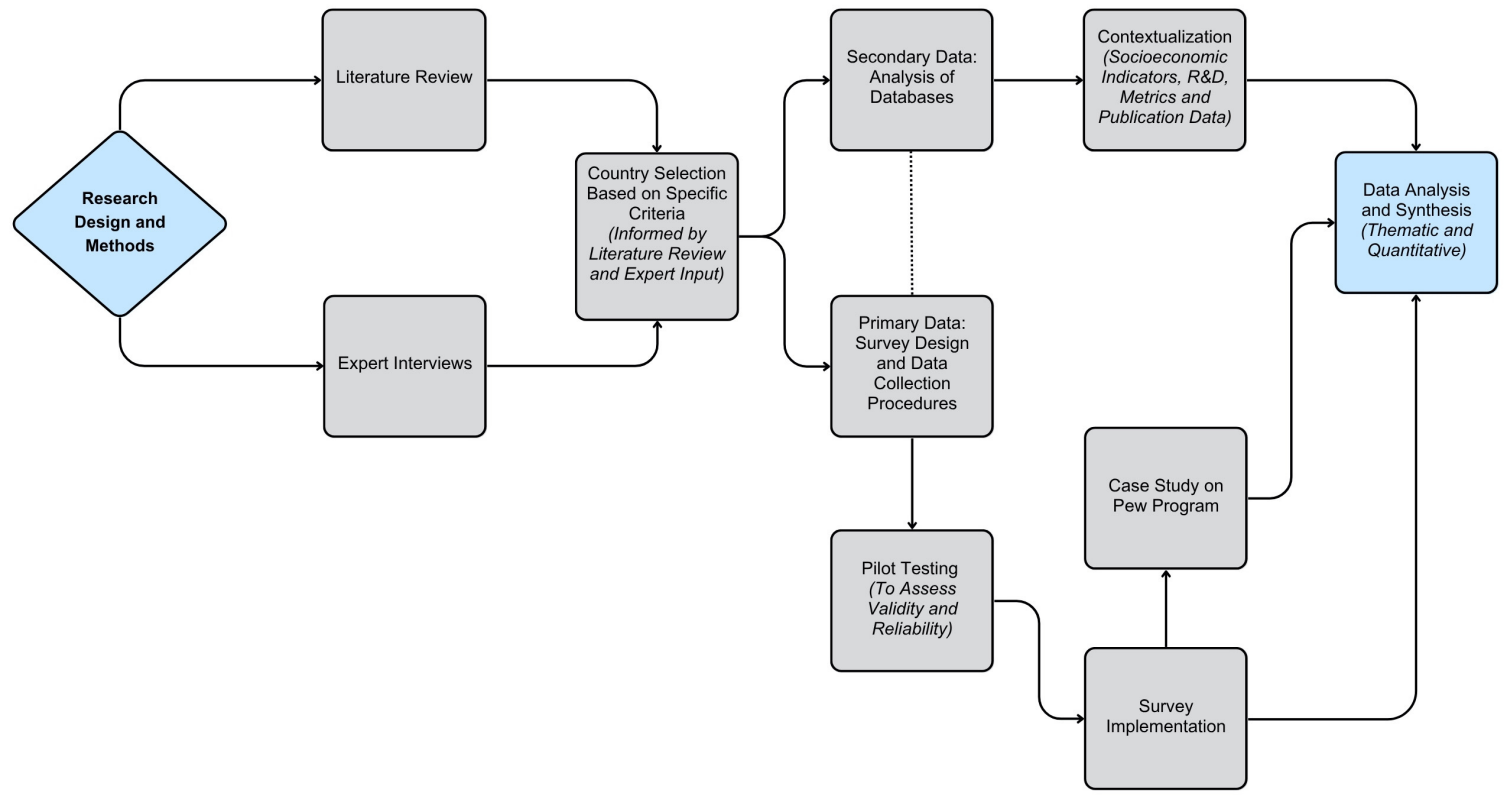
Flow chart of the key components of the research design and methods. This figure illustrates the sequential components of the research design and methods, including literature review, expert input, country selection, data collection, contextualization, and data analysis.

A multi-method approach was adopted to collect quantitative and qualitative data on biomedical research capacities in the selected countries. Figure 1 outlines the overall research design and serves as a visual guide to the sequential steps of the study, including the literature review, expert interviews, country selection, survey development and pilot testing; data collection through survey and case study; contextual analysis using socioeconomic, R&D, and publication indicators, and final data analysis and synthesis through both thematic and quantitative methods.

### Country selection and literature review

For our study, we selected five countries—Colombia, Peru, Guatemala, Costa Rica, and Panama—based on three primary criteria. First, these countries represent a middle tier of research performance within Latin America. They are positioned below the leading nations such as Brazil, Mexico, Chile, and Argentina, yet they demonstrate significant potential for growth. Second, the selection ensures geographical diversity by including countries from both Central America (Guatemala, Costa Rica, and Panama) and Andean subregion (Colombia and Peru), thereby offering a representative cross-section of scientific and cultural contexts. Third, these countries were chosen because they provided sufficient data on scientific output, socioeconomic indicators, and institutional infrastructure, which enabled a robust and meaningful analysis.

The selected countries exemplify varying stages of research infrastructure development and distinct institutional support mechanisms. This diversity provides a nuanced perspective on the challenges and opportunities associated with building biomedical research capacity in Latin America. Our methodological approach allows for a detailed exploration of emerging research environments and informs actionable strategies for enhancing the region's scientific contribution.

Our country selection process was informed by a targeted literature review that examined biomedical research capacities across Latin America. This review considered research performance, including publication quality and quantity, and areas of scientific excellence; geographic diversity; and data availability, specifically on scientific output, socioeconomic indicators, and institutional infrastructure. We conducted the review across databases such as PubMed, Scopus, Web of Science, and Google Scholar, and supplemented it with government and international organization reports. We prioritized peer-reviewed articles and credible reports from the past 20 years, using keywords and Boolean operators to ensure comprehensive coverage. A narrative synthesis integrated the findings, identifying recurring themes related to research strengths (e.g., infrastructure, policy frameworks), challenges (e.g., funding, personnel shortages), and opportunities (e.g., best practices, expert recommendations). Our transparent and documented search strategy prioritized high-quality, trustworthy sources, including relevant gray literature.

### Data collection methods

#### Interviews

Semi-structured interviews (Flick, 2018) were conducted with regional policy experts, academic researchers, and institutional leaders. These interviews, conducted prior to the survey, provided qualitative insights into systemic challenges, successful practices, and strategies for fostering improvement. They enriched the study's findings by uncovering context-specific barriers and opportunities that quantitative methods could not fully address.

This qualitative component involved interviews with 12 biomedical research experts from Latin American countries, conducted between March and June 2024. The participants included three each from Colombia, Uruguay, and Peru, and one each from Guatemala, Panama, and Costa Rica. A structured interview questionnaire guided the discussions, covering topics such as awareness of the Pew Latin American Fellows program, research environments, infrastructure, funding sources, and barriers to program participation.

Interviews were conducted in Spanish to ensure a comprehensive understanding of biomedical research capacities, challenges, and opportunities in the region. Detailed notes were taken during each session, and all but two interviews—at the interviewees' request—were audio-recorded. Professional transcription services were used to transcribe the recordings, and the research team reviewed the transcripts against the original recordings to ensure accuracy.

Thematic analysis was applied to the transcribed data through iterative coding, enabling the identification of key themes and patterns. The analysis synthesized these themes into a comprehensive summary of the most salient findings and trends.

Insights from the interviews informed the design of the broader survey used to collect quantitative data on biomedical research capacities. Additionally, they guided the selection of the five countries included in the study. This qualitative approach provided valuable context and depth, enhancing the overall robustness of the study.

#### Surveys

Building on the interviews, we distributed surveys in July and August 2024 to key professionals and stakeholders involved in biomedical research across these countries to collect quantitative data on institutional capacities, funding sources, educational opportunities, and researcher challenges. The survey aimed to capture a broad understanding of the research environment and identify structural and resource-related barriers impacting biomedical research.

A non-probability sampling method was employed to assess biomedical research capacities in the selected countries. The target population included professionals and stakeholders directly engaged in or significantly affected by biomedical research activities. The sampling frame comprised principal investigators, senior scientists, academic leaders (e.g., deans, department chairs, and research institute directors), institutions such as public and private universities with biomedical research programs, and public and independent research institutes focused on specific areas of biomedical research.

An email-based approach was used for survey distribution, enabling efficient data collection and broad participation from the biomedical research community. The survey was conducted primarily in Spanish, with an English version available for international participants or those preferring English communication. To ensure clarity and functionality, a pilot test was conducted with 14 respondents, whose feedback informed improvements to the survey. The pilot test lasted 2 weeks, followed by a 3-week data collection period for the main survey.

Given the non-probability sampling design and the chosen distribution method, data weighting was not applied. The total sample size was determined to balance representation of critical subgroups within the biomedical research community with the constraints of a limited study period and budget. This methodology offered a comprehensive assessment of biomedical research capacity, offering valuable insights to guide capacity-building initiatives and enhance participation in international research programs.

#### Case study analysis

The Pew Latin American Fellows Program in Biomedical Sciences was analyzed as a case study to examine its role in fostering research capacity across the selected countries. This analysis assessed program awareness, perceived relevance, and barriers to participation. Furthermore, the study explored how the program's outreach and resources could be optimized to maximize its impact on capacity-building efforts in the region. The case study approach allowed for a focused evaluation of how international initiatives contribute to developing biomedical research capacities in emerging scientific environments.

To examine the Pew Latin American Fellows Program's role in fostering biomedical research capacity across selected Latin American countries, we conducted a focused case study analysis assessing program awareness, perceived relevance, and barriers to participation. The analysis also explored how outreach strategies and available resources could be optimized to enhance the program's impact on regional capacity-building in emerging scientific environments.

To comprehensively assess the program's impact, we combined quantitative and qualitative methods. Specifically, we performed a quantitative analysis of program data, including fellowship distribution from 1991 to 2023. Additionally, to gain deeper insights into application trends and barriers to participation, we requested and obtained information directly from Pew program associates. This qualitative inquiry provided valuable context regarding application numbers, selection rates, and factors influencing the uneven distribution of fellowships across Latin American countries.

### Socioeconomic indicators and publication data analysis approach

To contextualize biomedical research capacities, the study analyzed key socioeconomic, R&D, and publication indicators.

#### Socioeconomic indicators

Socioeconomic data were analyzed to understand the broader environmental factors influencing biomedical research potential. Metrics such as GDP per capita, the Human Development Index (HDI), health expenditure, and education levels were collected from reliable sources, including the World Bank, the United Nations Development Program (UNDP), the Ibero-American Network of Science and Technology Indicators, and the World Health Organization (WHO). These indicators highlighted conditions shaping each country's ability to support biomedical research, offering essential context for evaluating research potential across the region.

#### Research and Development (R&D) indicators

R&D-specific metrics were examined to assess investment levels and human capital availability in biomedical research. Key indicators included R&D expenditure as a percentage of GDP and the number of researchers per labor force member. These metrics provided an understanding of the resources dedicated to research and the workforce's capacity to support innovation, highlighting disparities and opportunities for growth across countries.

#### Publication data

Publication data were analyzed to evaluate scientific productivity and collaboration trends in biomedical research. Data from SCImago Journal & Country Rank, a publicly available resource that ranks journals and countries based on scientific indicators, were used to examine patterns in scientific output, co-authorship networks, and citation metrics. These data were used in our analysis correlating them with R&D investments and the effectiveness of regional and international collaborations in advancing biomedical research.

#### Data analysis and synthesis

Thematic analysis was applied to qualitative interview data to identify key patterns, challenges, and opportunities across the selected countries. Quantitative survey data were analyzed descriptively to assess trends in research infrastructure, funding, training programs, and institutional capacities. Together, these methods provided an integrated understanding of biomedical research capacities, enabling the triangulation of findings and the formulation of evidence-based recommendations.

## Results

This section presents the key findings from the assessment of biomedical research capacities in Colombia, Peru, Guatemala, Costa Rica, and Panama. It focuses on socioeconomic factors, research infrastructure, institutional capacities, publication metrics, and insights from the Pew Latin American Fellows Program. The findings reflect an integrated analysis combining thematic insights from expert interviews with quantitative trends drawn from survey data and secondary indicators.

### Analysis of socioeconomic factors and publication metrics

This study examined major socioeconomic factors, research and development metrics, and publication data to establish the context for biomedical research capabilities. By integrating these data sources, the study provides a comprehensive understanding of the biomedical research landscape, highlighting systemic challenges and opportunities for targeted interventions.

#### Socioeconomic indicators

The analysis of socioeconomic indicators across the studied countries reveals critical insights into the regional biomedical research landscape (Table 1). Key indicators, including GDP per capita, Human Development Index (HDI), mean years of schooling, and health expenditure as a percentage of GDP, provide valuable insights into each country's strengths and challenges in fostering biomedical research ecosystems.

**Table 1:** Socio-economic development indicators for selected Latin American countries.

**Indicator**	**Colombia**	**Peru**	**Costa Rica**	**Panama**	**Guatemala**
Surface area (Square Km)	1,138,910	1,285,216	51,100	75,417	108,889
Population (2022)	51,874,024	34,049,588	5,180,829	4,408,581	17,357,886
GDP per capita (2022)	6,624.2	7,125.8	13,365.4	17,357.6	5,473.2
Human development index (2022)	0.758	0.762	0.806	0.820	0.629
Mean years of schooling (2022)	8.9	10.0	8.8	10.7	5.7
Life expectancy at birth (2020)	73	72	77	76	69
Mortality rate, infant, male (2022)	12	13	7	12	21
Adult literacy rate (%, 2022)	96	94	98	96	84
Poverty headcount ratio (%, 2022)	6.0	2.7	0.9	1.3	9.5
Current health expenditure (% of GDP, 2022)	9.02	5.10	7.57	9.68	6.90

Sources: Data for this table were sourced from the World Bank's World Development Indicators (retrieved June 25, 2024), the RICYT indicators (retrieved July 14, 2024, from https://www.ricyt.org/en/category/indicators/), the Human Development Index (retrieved July 14, 2024, from http://hdr.undp.org/en/composite/HDI), and the WHO Global Health Expenditure database (apps.who.int/nha/database). Note that literacy rate refers to the adult total percentage of those aged 15 and above. Poverty headcount ratio is measured at USD 2.15 per day, expressed as a percentage of the population. The mortality rate is for infant males per 1,000 live births (2022).

#### GDP per capita (2022)

Panama ($17,357.6) and Costa Rica ($13,365.4) lead in GDP per capita, offering a stronger economic foundation for R&D investment. In contrast, Peru ($7,125.8), Colombia ($6,624.2), and Guatemala ($5,473.2) have lower GDP per capita, indicating more limited resources for large-scale biomedical research.

#### Human development index (2022)

The HDI values for Panama (0.820) and Costa Rica (0.806), along with their life expectancy and education metrics, indicate high overall development. Colombia (0.758) and Peru (0.762) show moderate progress, while Guatemala (0.629), the lowest, faces systemic barriers limiting access to essential resources.

#### Mean years of schooling (2022)

Panama (10.7) and Peru (10.0) have the highest mean years of schooling, highlighting stronger educational attainment that contributes to a skilled workforce. Colombia (8.9) and Costa Rica (8.8) are slightly lower, reflecting moderate levels of educational attainment. Guatemala (5.7), however, has the lowest mean years of schooling.

#### Health expenditure (% of GDP, 2024)

Panama (9.68%) and Colombia (9.02%) demonstrate strong public health investment, which is relevant for biomedical research. Costa Rica (7.57%) follows, prioritizing health systems, while Guatemala (6.90%) and Peru (5.10%) allocate significantly less.

### Research and Development (R&D) as a percentage of Gross Domestic Product (GDP)

**Figure 2:**
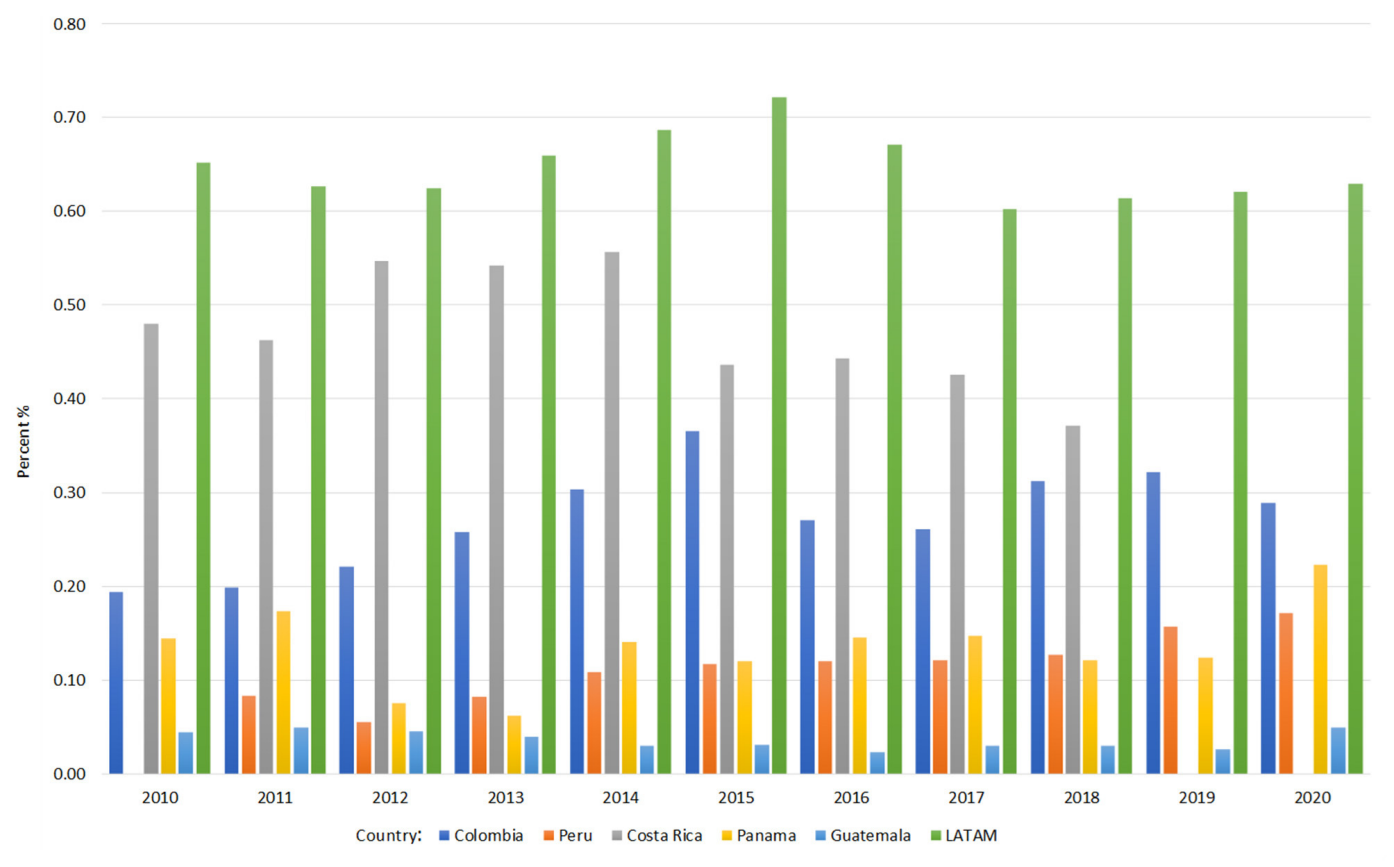
Government expenditure on Research and Development (R&D) as a percentage of GDP. This figure illustrates the trends in government expenditure on Research and Development (R&D) as a percentage of Gross Domestic Product (GDP) for selected Latin American countries from 2010 to 2020.

Trends in government R&D expenditure (as a percentage of GDP) for selected Latin American countries between 2010 and 2020 are illustrated in Figure 2.

Across all five countries, R&D expenditure as a percentage of GDP remains below global averages, typically ranging between 0.1% and 0.5%. Costa Rica ranks among the highest in the group, with R&D expenditure nearing 0.4%−0.5% of GDP during the analyzed period. Colombia similarly demonstrates a modest yet steady commitment, averaging around 0.3%−0.4% of GDP from 2010 to 2020. In contrast, Panama maintains one of the lowest levels of R&D investment, consistently below 0.2% of GDP, while Guatemala reports the lowest expenditure, often below 0.1% of GDP. Peru's investment patterns remain variable, fluctuating between 0.2% and 0.3% of GDP. While Costa Rica and Colombia invest more in R&D than their regional counterparts, their allocations remain well below the global benchmark of 2%−3% and even Brazil's 1.2% of GDP.

### Evolution of research capacity in selected Latin American countries (2012–2021)

A comparative analysis of research capacity and innovation output across selected Latin American countries reveals notable variations in scientific capacity (Table 2). The study examined key metrics, including researcher density, per capita R&D expenditure, and patent applications by residents and non-residents for the years 2012, 2016, and 2021.

**Table 2:** Research workforce, R&D investment per capita, and patent activity (2012–2021).

**Country**	**Number of researchers per 1,000 labor force (HC)**			**R&D Expenditure on S&T per capita (USD)**			**Patent applications by residents (per million inhabitants)**			**Patent applications by non-residents (per million inhabitants)**		
	**2012**	**2016**	**2021**	**2012**	**2016**	**2021**	**2012**	**2016**	**2021**	**2012**	**2016**	**2021**
Brazil	2.67	3.68		140.07	112.59		7,795	8,123	7,288	25,744	22,938	19,633
Mexico	0.81	1.01	1.16	43.24	34.08	27.87	1,292	1,310	1,117	14,022	16,103	15,044
Argentina	4.67	4.89	4.88	88.46	71.39	55.06	697	854	406	4,119	2,953	3,263
Chile	1.28	1.63		50.03	43.74		336	386	399	2,683	2,521	2,683
Colombia		0.53	0.91	18.83	14.94	12.45	206	507		2,022	1,703	
Peru	0.09	0.25	0.49	3.66	7.57	9.42	51	68	95	1,135	1,090	1,142
Costa Rica	1.66	1.70	1.86	55.34	53.35	34.46	37	44		631	545	
Panama	0.26	0.28	0.58	8.02	19.01	27.22		68	35	234	349	
Guatemala	0.11	0.10	0.06	1.52	0.96	2.95	7	4		350	278	

Sources: Data for this table are from RICYT (Network for Science and Technology Indicators—Ibero-American and Inter-American), retrieved April 25, 2024, from https://www.ricyt.org/en/category/indicators/. The “Researchers per 1,000 Labor Force” metric represents the number of natural persons engaged in research per 1,000 members of the labor force. “R&D Expenditure on S&T per Capita” indicates the Research and Development expenditure on Science and Technology, measured in current US dollars per capita.

The number of researchers per 1,000 labor force reflects the availability of skilled professionals in research. Costa Rica maintained steady progress, rising from 1.66 in 2012 to 1.86 in 2021, demonstrating sustained investment. Colombia showed improvement, rising from 0.53 in 2016 to 0.91 in 2021, indicating a strengthening research workforce. Peru experienced significant growth, increasing from 0.09 in 2012 to 0.49 in 2021, despite starting from a low base. Panama also exhibited growth, from 0.26 in 2012 to 0.58 in 2021, reflecting an emerging commitment to expanding research personnel. However, Guatemala showed a decline, dropping from 0.11 in 2012 to 0.06 in 2021, highlighting difficulties in attracting and retaining researchers.

R&D per capita expenditure on science and technology (S&T) is a crucial indicator of research capacity. Costa Rica experienced a decline, from $55.34 in 2012 to $34.46 in 2021, possibly due to budget constraints. Panama showed consistent growth, rising from $8.02 in 2012 to $27.22 in 2021. Peru exhibited modest growth, increasing from $3.66 in 2012 to $9.42 in 2021, though it remained among the lowest. Colombia saw a decrease from $18.83 in 2012 to $12.45 in 2021. Guatemala maintained the lowest investment level among the studied countries, with per capita R&D spending of just $2.95 in 2021, up slightly from $1.52 in 2012.

Patent applications by residents, a measure of a country's innovation output, showed varied results. Colombia experienced substantial growth, from 206 in 2012 to 507 in 2016, indicating progress in fostering innovation. Peru demonstrated modest growth, increasing from 51 in 2012 to 95 in 2021, signaling slow but steady improvement. Costa Rica remained relatively low, increasing slightly from 37 in 2012 to 44 in 2016, with no data for 2021. Panama showed progress, from 68 in 2016 to 35 in 2021, suggesting emerging innovation potential. Guatemala had consistently low numbers, with no data for 2021.

Patent applications by non-residents reflect international collaboration and interest. Colombia saw a decline, from 2,022 in 2012 to 1,703 in 2016, possibly indicating reduced foreign engagement. Costa Rica exhibited fluctuations, decreasing from 631 in 2012 to 545 in 2016, which may suggest reduced external patent activity. Panama showed slight growth, from 234 in 2012 to 349 in 2016, reflecting increased external interest. Peru remained stable, fluctuating between 1,135 and 1,142, indicating consistent international involvement. Guatemala experienced a decline, from 350 in 2012 to 278 in 2016.

### Publication trends in biomedical scientific publications (1996–2023)

The analysis of biomedical publications across selected Latin American countries from 1996 to 2023 reveals significant variation in scientific productivity, with notable growth in several countries. These findings highlight disparities in scientific output that reflect underlying capacity gaps—an issue central to this study's objective of assessing biomedical research capacity in the region.

#### Cumulative biomedical publications (1996–2023)

Using SCImago Journal & Country Rank (Souza et al., 2019), the data are categorized into three primary biomedical fields: (1) Biochemistry, Genetics and Molecular Biology, (2) Immunology and Microbiology, and (3) Neurosciences. Key metrics for each country include total scientific publications, citable documents, citations, and self-citations. This analysis highlights the region's diverse research outputs and capacities, offering insights into strengths and opportunities for collaboration and growth.

Table 3 illustrates cumulative data on scientific publications, citable documents, citations, and self-citations. Brazil leads by a substantial margin, with 252,569 publications and nearly 6 million citations, reflecting its dominant position in Latin American biomedical research. Mexico (83,530 publications) and Argentina (65,963 publications) follow, maintaining strong outputs alongside high citation counts. Chile ranks fourth, with over 33,000 publications, showcasing steady growth in recent years.

**Table 3:** Cumulative scientific publications in biomedical areas by selected countries, 1996–2023.

**Country**	**LATAM ranking/H-index**			**Total scientific publications**	**Citable documents**	**Citations**	**Self-citations**
	**Biochemistry, genetics, and molecular biology**	**Immunology and microbiology**	**Neuroscience**				
Brazil	1 (388)	1 (279)	1 (250)	252,569	243,216	5,919,720	1,563,660
Mexico	2 (318)	2 (230)	2 (163)	83,530	80,629	2,102,336	527,547
Argentina	3 (296)	3 (205)	3 (174)	65,963	63,818	18,38,131	280,642
Chile	4 (246)	4 (146)	4 (143)	32,058	33,184	995,633	124,317
Colombia	5 (182)	5 (137)	5 (103)	25,933	22,545	496,354	63,236
Peru	9 (136)	8 (107)	9 (60)	7,990	7,607	192,589	18,609
Costa Rica	12 (133)	11 (80)	12 (54)	3,727	3,626	125,332	10,591
Panama	13 (137)	13 (80)	13 (53)	2,584	2,781	125,472	9,631
Guatemala	17 (58)	17 (50)	21 (16)	815	779	22,614	1,366

Documents Published, Citable Documents, Citations, and Self-Citations.

Sources: Data for this table were sourced from SCImago, specifically the SJR—SCImago Journal & Country Rank, retrieved April 18, 2024, from http://www.scimagojr.com. Note: data for Neuroscience are unavailable for the following years and countries: Costa Rica in 1999; Panama in 1999, 2003, and 2005; and Guatemala in 1997 through 1999, 2001 through 2003, 2006, and 2010 through 2011.

In contrast, countries such as Colombia (25,933 publications), Peru (7,990), Costa Rica (3,727), Panama (2,584), and Guatemala (815) demonstrate significantly lower outputs. Notably, Colombia shows promising progress, outperforming other middle-tier countries in both publication volume and citations. Guatemala, with the smallest output, reflects persistent challenges in research infrastructure and funding.

#### Trends in biomedical scientific publications (1996–2023)

**Figure 3:**
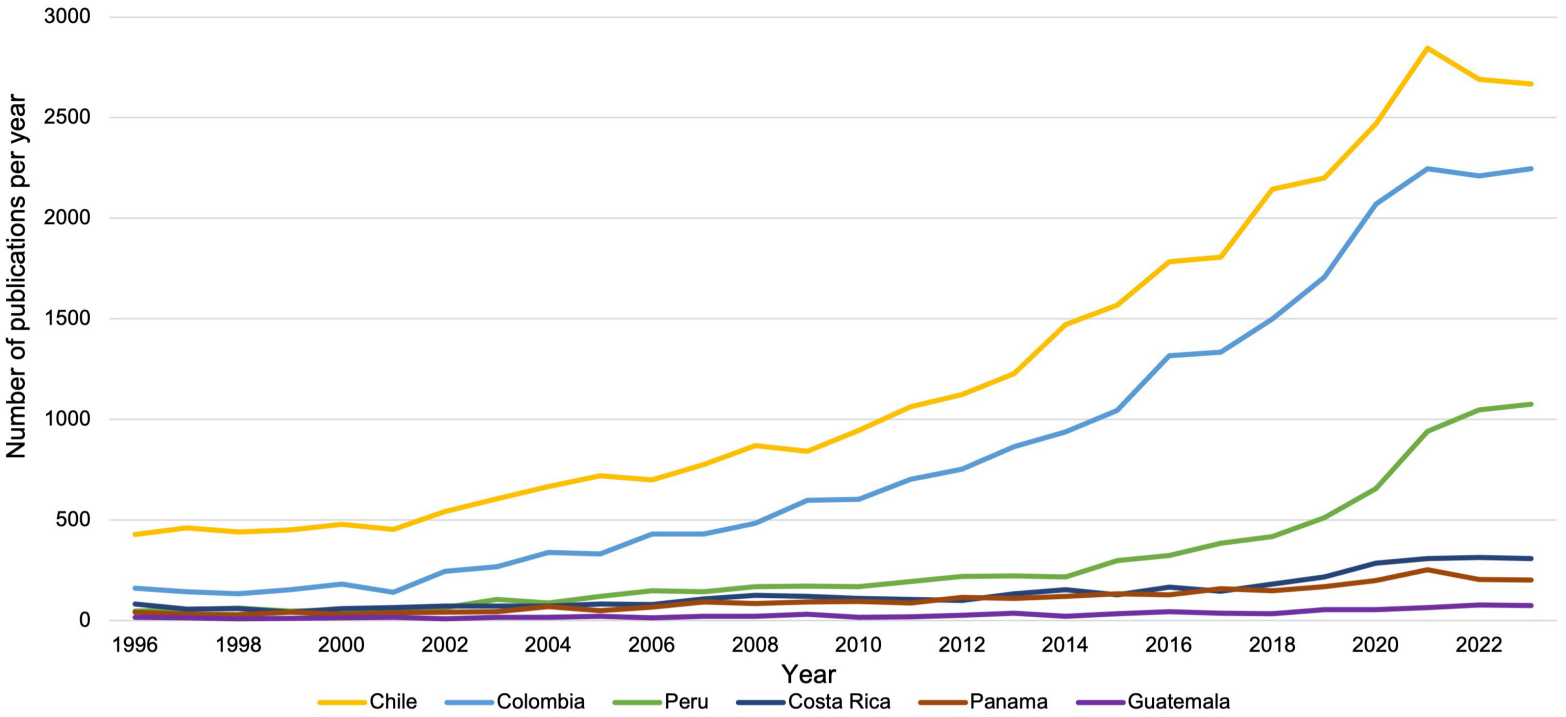
Trends in biomedical research publications (1996–2023). This figure shows the annual number of biomedical research publications from selected Latin American countries between 1996 and 2023, highlighting trends in scientific output over time.

To compare trends among the selected countries, we analyzed yearly publication data on biomedical research, combining data from three primary fields, as detailed in Table 3: (1) Biochemistry, Genetics, and Molecular Biology; (2) Immunology and Microbiology; and (3) Neurosciences. Figure 3 illustrates an overall upward trend in regional biomedical research, with growth accelerating after the early 2000s. Among the middle-tier countries, Colombia has demonstrated the strongest recent growth, while Peru, Costa Rica, and Panama have experienced modest but steady increases. Guatemala's publication output has remained largely stagnant. Chile is included in Figure 3 as a regional reference. Although Brazil and Mexico saw sharp increases post-2010, they, along with Argentina, were excluded from the figure for clarity.

### Institutional capacities and support systems

**Figure 4:**
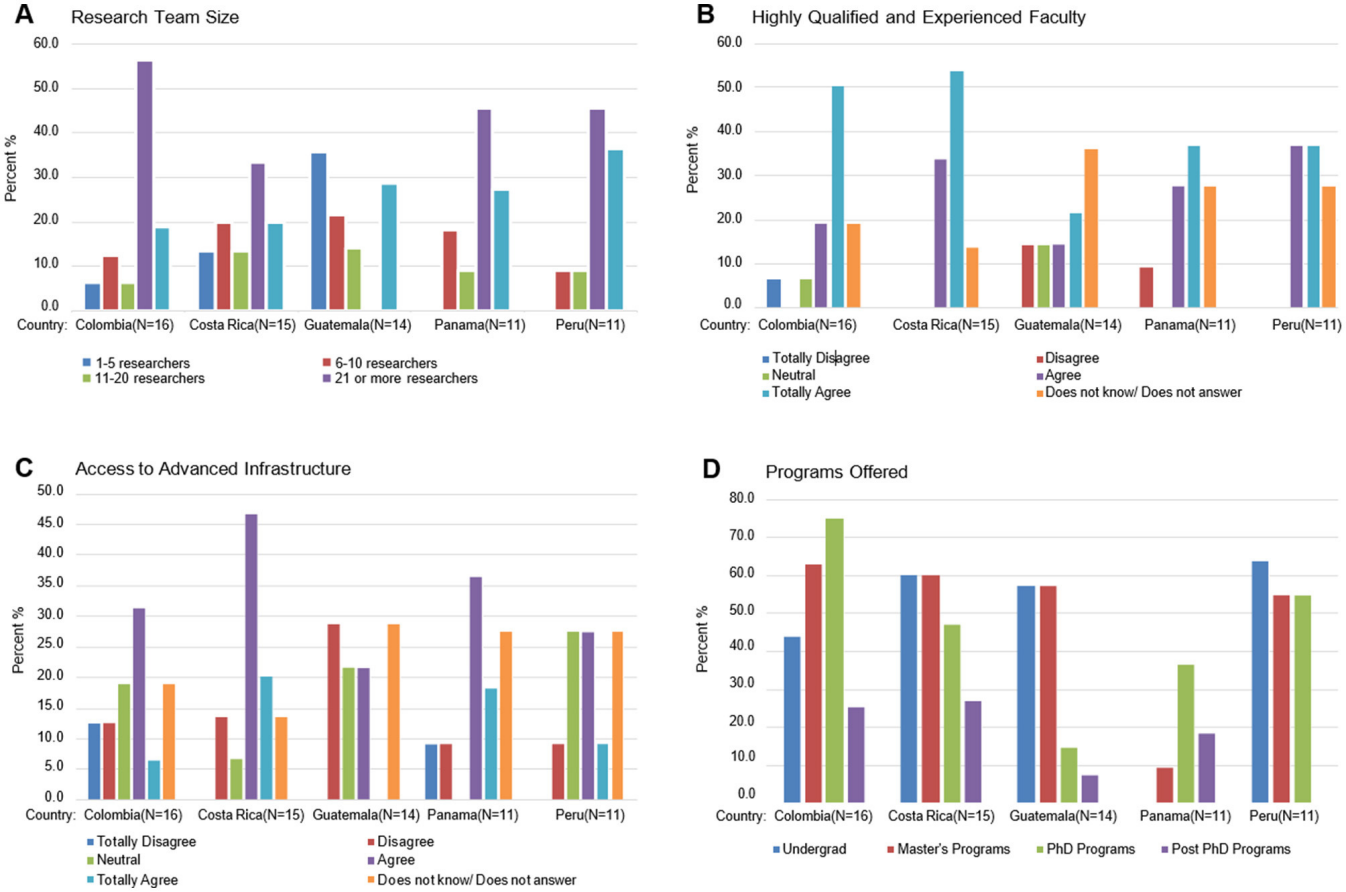
Institutional capacities and resources. **(A)** Research team size; **(B)** highly qualified and experience faculty; **(C)** access to advanced infrastructure; **(D)** programs offered.

This section examines the institutional landscapes supporting biomedical research across the selected countries, synthesizing survey responses related to research capacities, specialization areas, faculty qualifications, infrastructure access, and funding support (Figure 4).

#### Research capacities and team dynamics

The study found significant variations in research team sizes across institutions in the five surveyed countries, indicating disparities in research capacity (Figure 4A). Colombia had the strongest research capacities, with 56.3% of respondents reporting institutions hosting teams of 21 or more researchers. Panama and Peru followed, both with 45.5% of respondents reporting similarly sized teams. Guatemala, in contrast, had smaller team structures, with 35.7% of institutions reporting teams of 1–5 researchers, suggesting limited research capacity. Medium-sized teams (6–10 or 11–20 researchers) were common in Costa Rica, Panama, and Peru.

#### Specialization areas

The survey uncovered diverse specialization patterns across the participating institutions, reflecting each country's unique research priorities and strengths. In Colombia, Immunology and Microbiology/Parasitology were the most prominent fields, each cited by 68.75% of institutions. Costa Rica exhibited a strong focus on Genetics (66.67%), followed by Immunology and Cellular Biology, both represented in 53.33% of institutions. These fields indicate a growing interest in foundational biomedical research.

In contrast, Guatemala demonstrated a more diversified research landscape. Genetics emerged as the leading area of specialization (28.57%), with Immunology and Microbiology/Parasitology equally represented (21.3%). Panama, however, stood out for its emphasis on technologically advanced fields, such as Biotechnology (72.73%) and Bioinformatics/Computational Biology (63.64%). These trends suggest Panama's strategic focus on leveraging emerging technologies for biomedical advancements. In Peru, Microbiology/Parasitology was the dominant specialization area (63.64%), reflecting a focus on addressing infectious diseases, while Biochemistry, Biotechnology and Cellular Biology followed at 54.55%, indicating an emphasis on molecular-level research.

#### Faculty qualifications

Faculty expertise was highly regarded in Colombia and Costa Rica (Figure 4B), where over 50% of respondents affirmed the presence of highly qualified and experienced research faculty. In contrast, Peru, Panama, and Guatemala showed more mixed perceptions, with higher levels of neutrality and uncertainty in responses. While some respondents acknowledged faculty expertise, the prevalence of neutral and non-committal answers.

#### Access to advanced infrastructure

The survey showed varying levels of access to advanced biomedical research facilities (Figure 4C). Costa Rica had the most positive response, with 66.7% of respondents reporting adequate access (46.7% agreed, 20.0% strongly agreed). Panama (54.6% total agreement−36.4% agreed and 18.2% strongly agreed) and Colombia (37.6% total agreement−31.3% agreed and 6.3% strongly agreed) showed moderate agreement. Peru reflected mixed perceptions; while 36.4% agreed (27.3% agreed and 9.1% strongly agreed), a notable percentage (27.3%) remained neutral or uncertain. Guatemala had the highest disagreement rate (28.6%), indicating more limited access.

#### Interdisciplinary research collaboration

Support for interdisciplinary research collaboration varied significantly across countries. Costa Rica led with 60% of respondents affirming that their institutions foster collaboration (33.3% agree, 26.7% totally agree), followed by Guatemala (57.2%: 42.9% agree, 14.3% totally agree) and Colombia (43.8%: 25.0% agree, 18.8% totally agree). Panama showed a divided perception, with 27.3% disagreeing and an equal percentage remaining neutral, indicating inconsistencies across institutions. Peru exhibited moderate support, with only 27.3% agreement and a high level of neutrality (36.4%), suggesting uncertainty or lack of institutional commitment to interdisciplinary collaboration.

#### Support for biomedical research funding

The survey results provide valuable insights into institutional support for biomedical research funding across the studied countries. Two key areas were analyzed: institutional funding opportunities, and grants and support to secure external funding.

The most institutional funding opportunities were perceived by respondents in Colombia (50%), while those in Panama reported the least (54.6% disagreement). Elsewhere, perceptions were mixed in Peru (36.4% disagreed), weak in Guatemala (21.4% agreed), and largely neutral in Costa Rica (40%).

Perceptions of external funding opportunities varied. Colombia (68.8%) and Peru (54.6%) reported the most support, Guatemala (14.3%) the least. Panama (45.5%) and Costa Rica (26.7%) had mixed results, with significant neutrality.

#### Sources of funding for biomedical research

Survey results reveal diverse funding patterns across the five countries studied. Government funding dominates in Colombia (81.3%), Panama (72.7%), and Peru (63.6%), with lower levels in Costa Rica (46.7%) and Guatemala (21.4%). Private sector investment is highest in Guatemala (42.9%), minimal in Colombia (6.3%) and Costa Rica (20.0%), and absent in Panama and Peru. International agencies provide substantial support to Colombia (43.8%), Guatemala (35.7%), Panama (36.4%), and Peru (36.4%), but none to Costa Rica. NGO funding exists in Costa Rica (13.3%), Guatemala (21.4%), and Peru (18.2%), while institutional funds contribute modestly in Costa Rica (6.7%), Guatemala (14.3%), and Colombia (6.3%). Both NGO and institutional funding are notably absent in Panama.

### Training programs and early-career support

The uneven availability of training programs and mentorship opportunities underscores systemic barriers to workforce development in the region, limiting the ability of national institutions to train, retain, and advance the next generation of biomedical researchers—a central concern in strengthening long-term research capacity.

### Training programs in biomedical sciences

The survey on biomedical sciences training programs revealed significant variation across the five surveyed countries at various academic levels (Figure 4D). These results point to disparities in access to training and mentorship opportunities, highlighting systemic barriers to workforce development and their potential impact on research capacity in the region.

#### Undergraduate programs

Costa Rica (60.0%) and Peru (63.6%) show a strong commitment to undergraduate biomedical education, suggesting a robust pipeline for future researchers. Colombia (43.8%) and Guatemala (57.1%) also offer undergraduate programs. In contrast, Panama reports no undergraduate programs (0.0%), highlighting a critical gap in foundational training.

#### Master's programs

Master's programs are relatively well-established across most countries, with Colombia (62.5%), Costa Rica (60.0%), and Guatemala (57.1%) leading. Peru (54.5%) also performs well, suggesting a strong focus on advanced education in the region. However, Panama (9.1%) lags significantly, reporting limited opportunities for specialized training.

#### PhD programs

Doctoral programs are most prevalent in Colombia (75.0%), followed by Peru (54.5%), indicating a conducive environment for advanced research training. Costa Rica (46.7%) and Panama (36.4%) offer moderate levels of PhD programs, while Guatemala (14.3%) lags significantly.

#### Postdoctoral programs

Postdoctoral opportunities are scarce across all surveyed countries. Peru reports none (0.0%), and even in Costa Rica (26.7%) and Colombia (25.0%), availability is only slightly higher. Guatemala and Panama also appear to have limited opportunities.

Additional programs reflect further differences and unique institutional approaches. Colombia provides a diverse range of educational opportunities, including diplomas and specializations, while Panama focuses on mentoring for thesis projects. Peru offers unpaid internships, which, despite their potential value, raise concerns about accessibility and equity.

#### Professional development

The emphasis on mentorship and professional development varied significantly across countries. Colombia received the most positive feedback, with 43.8% of respondents expressing satisfaction (31.3% agreed and 12.5% strongly agreed), followed by Peru, with 36.4% agreeing, and Costa Rica, with 33.4% (6.7% agreed and 26.7% strongly agreed). Panama presented a mixed picture across the scale of responses. Uncertainty was also prevalent, with 28.6% of respondents in Guatemala and 27.3% in both Panama and Peru unsure about their institutions' focus on this area.

### Support for students and early-career researchers

**Figure 5:**
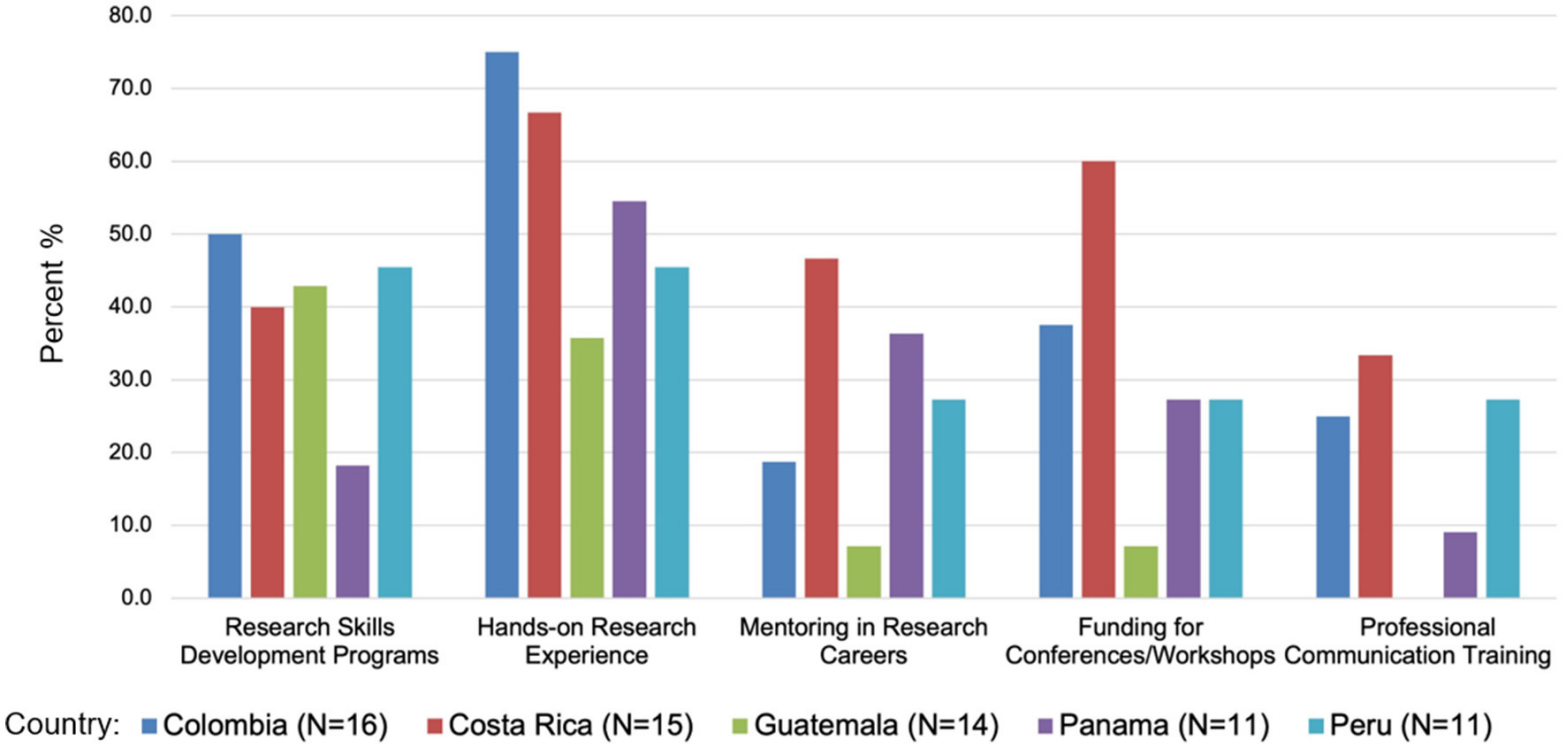
Support for students and early-career researchers. This figure illustrates the variation in support systems for students and early-career researchers across the studied countries, including research skills development programs, hands-on research experience, mentoring in research careers, funding for conferences and workshops, and professional communication training.

The survey revealed significant variability in the support systems available for students and early-career researchers across the studied countries (Figure 5). Colombia, Costa Rica, and Peru emerged as leaders in providing resources, while Guatemala and Panama demonstrated notable gaps.

Programs to enhance research skills, such as experimental design and data analysis, were most prevalent in Colombia (50%) and Peru (45.5%), followed closely by Guatemala (42.9%) and Costa Rica (40%). Panama, however, showed the lowest percentage (18.2%).

Opportunities such as internships and rotations were a key focus in Colombia (75%) and Costa Rica (66.7%), demonstrating a commitment to providing hands-on experience for early-career researchers. Panama (54.5%) and Peru (45.5%) also offered moderate levels of such opportunities, whereas Guatemala lagged significantly, with only 35.7% of respondents reporting such resources.

Mentoring or career guidance programs showed substantial variation, with Costa Rica leading at 46.7%, followed by Panama (36.4%) and Peru (27.3%). Colombia (18.8%) and Guatemala (7.1%) reported notably lower percentages.

Financial support for attending conferences or workshops was a strength in Costa Rica (60%), setting it apart as the leader in this category. Colombia, Panama, and Peru provided more limited support, with rates ranging from 27.3% to 37.5%, while Guatemala demonstrated the lowest levels, at only 7.1%.

Training in professional communication skills was the least emphasized area across all countries, with Costa Rica (33.3%) and Peru (27.3%) showing moderate efforts. Colombia (25%) and Panama (9.1%) provided minimal focus on such workshops, and Guatemala reported no such resources available.

### Major obstacles to successful biomedical research

**Figure 6:**
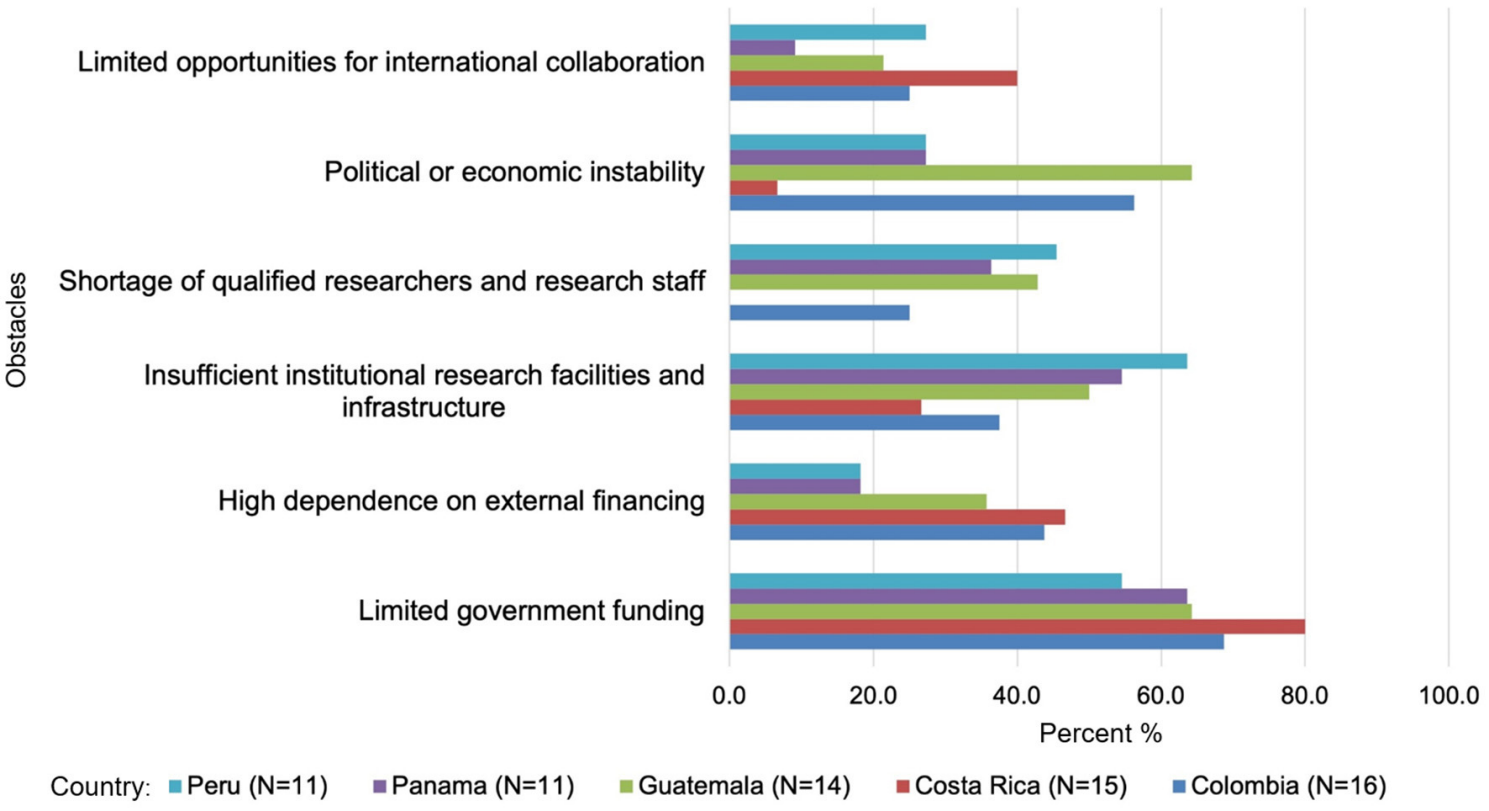
Major obstacles to successful biomedical research. This figure illustrates the main challenges hindering biomedical research in selected Latin American countries: funding limitations, inadequate infrastructure, personnel shortages, instability, and limited collaboration.

The survey identified several critical challenges affecting biomedical research in the studied countries. These include limited government funding, dependence on external financing, inadequate infrastructure, shortages of qualified personnel, political, and economic instability, and limited international collaboration (Figure 6).

#### Limited government funding

Limited government funding emerged as the most pressing issue across all countries, with Costa Rica (80%) reporting the highest concern, followed by Colombia (68.8%), Guatemala (64.3%), Panama (63.6%), and Peru (54.5%). These findings highlight a structural weakness in national research systems, where insufficient domestic investment undermines long-term capacity-building in biomedical science.

#### Dependence on external financing

High dependence on external financing further complicates the funding landscape, particularly in Costa Rica (46.7%) and Colombia (43.8%), where researchers rely heavily on inconsistent and short-term funding from international sources. In Guatemala (35.7%), Panama (18.2%), and Peru (18.2%), this reliance is somewhat less pronounced but still problematic, limiting the stability and continuity of research projects. This dependency reinforces the fragility of national research ecosystems and underscores the need for more sustainable and autonomous funding mechanisms.

#### Inadequate infrastructure

The lack of sufficient institutional research facilities and infrastructure was a prominent issue in Peru (63.6%), Panama (54.5%), and Guatemala (50%), significantly affecting the quality and scope of research. Colombia (37.5%) and Costa Rica (26.7%) reported fewer concerns, but the challenge remains substantial.

#### Shortages of qualified researchers

A shortage of qualified researchers and research staff is a critical bottleneck, especially in Peru (45.5%) and Guatemala (42.9%), where the lack of human capital hampers research timelines and reduces the diversity of expertise available. Panama (36.4%) and Colombia (25%) also face notable challenges, whereas Costa Rica (0%) reported no concerns in this area.

#### Political and economic instability

Political and economic instability was particularly concerning in Guatemala (64.3%) and Colombia (56.3%), where shifts in government priorities and economic uncertainties disrupt funding and research activities. Panama (27.3%), Peru (27.3%), and Costa Rica (6.7%) were less affected, though the issue remains a latent threat to long-term research commitments in these countries.

#### Limited international collaboration

While less pronounced than other challenges, limited opportunities for international collaboration were reported by Costa Rica (40%), Colombia (25%), Peru (27.3%), Guatemala (21.4%), and Panama (9.1%). This limitation reduces access to cutting-edge developments, global research networks, and international funding opportunities, ultimately hindering innovation.

These challenges illustrate persistent limitations in funding, infrastructure, and collaboration that directly constrain the region's ability to build and sustain robust biomedical research ecosystems. Addressing these systemic barriers is essential for advancing long-term capacity-building, fostering innovation, and enabling meaningful participation in global scientific networks.

### Research insights for the Pew Latin American fellows program

This analysis explores key findings from survey responses and expert interviews, examining interests and obstacles related to postdoctoral training and broader reflections on biomedical research capacity in Latin America. The section begins with interest, awareness, and barriers to program participation, followed by an overview of strengths and challenges in the region's research landscape. Key obstacles to participation are summarized in Table 4.

**Table 4:** Summary of obstacles preventing potential applicants from applying to the pew program, with representative interview quotes.

**Country**	**Key obstacles**	**Participant quotes**
Colombia	Language and Cultural Barriers: Limited English proficiency and low cultural value of postdoctoral training.	“*Limited English is a big issue. Many qualified people don't apply because they struggle with the language*.”
Guatemala	Few People with PhD Degrees: No PhD programs in the country; limited interest in postdoctoral studies. Non-Attractive Return Conditions: Few professional opportunities upon return, mostly contract-based teaching positions.	“*There are very few doctors from Guatemala interested in postdocs in the U.S. It's very difficult to find someone who meets all the criteria.” “If you return to Guatemala, the best job you'll get is teaching, and even then, there are no permanent positions*.”
Peru	Few People with PhD Degrees: Lack of PhD programs and general misunderstanding of PhD education's value.	“*There is no understanding of what a PhD is in Peru. We are not preparing enough professional staff at that level*.”
Panama	Few People with PhD Degrees: Limited number of PhD graduates and insufficient postdoctoral opportunities.	“*We don't have enough PhD graduates, and those who complete their studies often don't find good postdoc opportunities*.”
Costa Rica	Few People with PhD Degrees and Limited English Proficiency: Small pool of PhD graduates and language barriers restricting applications abroad.	“*The pool of candidates is small, and language barriers further limit potential applications to international programs*.”

#### Interest in postdoctoral training

Survey results indicate varying levels of interest in pursuing postdoctoral training abroad among researchers from the selected Latin American countries. Colombia showed the highest interest, with 68.8% of respondents expressing a strong inclination toward international postdoctoral opportunities, followed by Costa Rica (53.3%) and Panama (45.5%), reflecting moderate interest. In contrast, Guatemala (21.4%) and Peru (36.4%) reported lower enthusiasm, with a notable proportion in Guatemala (28.6%) and Panama (27.3%) expressing uncertainty. These findings are further explored in the Discussion, with specific attention to how programmatic improvements and institutional support could expand the reach and impact of international fellowships in the region.

#### Awareness of the Pew program

Awareness of the Pew Program remains limited across all surveyed countries. A striking 75% of Colombian respondents, 66.7% of Costa Rican respondents, and 71.4% of Guatemalan respondents reported that they had never heard of the program. Similarly, over 60% of respondents in Panama and nearly 45.5% in Peru were also unaware of the program's existence. Notably, no respondents reported being “very familiar” with the program, highlighting a critical gap in outreach and visibility that may be limiting application rates.

#### Barriers to participation

Interviews and surveys revealed several key barriers limiting participation in postdoctoral training abroad. Financial constraints were a major obstacle in Colombia (75% of survey respondents) and Guatemala (71.4%). Language barriers, particularly English proficiency, were significant in Colombia (62.5%) and Peru (54.5%), with many potential candidates feeling unprepared for English-speaking programs. Limited information about opportunities was a challenge, especially in Costa Rica (60%) and Peru (54.5%). Guatemala faces structural challenges, including a small pool of PhD graduates and limited doctoral programs. Family obligations were frequently cited as constraints in Panama (45.5%), Peru (45.5%), Colombia (43.75%), and Costa Rica (40%). In Costa Rica, despite a strong academic tradition, language barriers and a limited number of PhD graduates were key challenges. Panama also faces barriers related to a lack of postdoctoral training opportunities, which reduces awareness and interest in international fellowships.

Interviewees highlighted that the lack of institutional support for identifying and applying to international programs further exacerbates these barriers. Some participants also noted cultural factors, such as the undervaluation of postdoctoral training in certain academic environments, as contributing to low application rates for the Pew Program specifically. The key obstacles to participation are summarized in Table 4.

These findings are further examined in the Discussion, with particular attention to how targeted programmatic improvements and stronger institutional support could help broaden access to international fellowships and enhance their impact across the region.

### Broader reflections on biomedical research capacity

#### Strengths of biomedical research

Interviewees highlighted several strengths within the biomedical research landscape across the participating countries. Costa Rica was recognized for its strong academic foundation in biology, biochemistry, and medicine, with notable faculty qualifications and a culture of interdisciplinary collaboration. Colombia demonstrated strengths in applied biomedical sciences, particularly through partnerships with biotechnology and engineering faculties. Panama benefits from its strategic geographical location and emerging initiatives in biosciences, while Guatemala leverages strong international collaborations, especially with institutions in the United States. Peru was noted for its robust theoretical training at the undergraduate level, providing a solid foundation for advanced research.

#### Challenges hindering research growth

Despite notable strengths, significant challenges continue to hinder biomedical research development across the region, according to interviewees. A heavy reliance on state funding limits the scope and sustainability of research, particularly in Costa Rica and Guatemala, where funding is often tied to short-term, locally focused projects. Colombia faces obstacles related to obsolete research equipment and limited access to cutting-edge technologies, restricting its global competitiveness. Peru struggles with the lack of professionalization in scientific careers and a centralized research infrastructure, leaving regions outside the capital underserved. In Panama, logistical barriers, particularly regarding the handling and access to biological samples, further delay research progress.

Together, these findings provide a comprehensive portrait of biomedical research capacity in the region—highlighting both structural limitations and strategic strengths that inform the following discussion on implications, opportunities, and policy directions.

## Discussion

This study set out to assess biomedical research capacities in five Latin American countries—Colombia, Costa Rica, Guatemala, Panama, and Peru—by examining infrastructure, training, funding, and international engagement. There are five significant findings from our study, each highlighting key strengths and persistent challenges within the biomedical research landscape of the selected Latin American countries. Strategic investments are needed to address research capacity deficiencies and foster sustainable scientific growth, as highlighted by these findings. The discussion below interprets these findings in light of the study's objectives, identifying structural barriers, regional assets, and implications for science policy and capacity-building.

1) **Socioeconomics shape research capacity in the region**. Costa Rica and Panama demonstrate stronger human development and socioeconomic indicators, including higher GDP per capita, HDI, and life expectancy (Table 1). However, R&D expenditure (as a percentage of GDP) remains below global benchmarks across all five countries. Costa Rica's R&D expenditure neared 0.4%−0.5% of GDP (Figure 2), while Colombia averaged 0.3%−0.4% from 2010 to 2020. Costa Rica's per capita R&D expenditure on S&T currently averages $47.71, a level comparable to Chile, a leading country in the region (Table 2). In terms of researchers per 1,000 labor force, Costa Rica showed steady growth (1.66 in 2012 to 1.86 in 2021), as did Colombia (0.53 in 2016 to 0.91 in 2021). Guatemala and Peru lagged in these socioeconomic and research capacity metrics.
2) **Colombia exhibits growing leadership in biomedical publications among the top five countries**. Trends in biomedical scientific publications from 1996 to 2023 show that Colombia has established itself as a leader among middle-tier Latin American countries, demonstrating significant growth and potential, possibly reflecting successful international collaborations, as highlighted in the Results section on publication trends (Figure 3). While Peru and Costa Rica show moderate progress, Panama and Guatemala lag behind, underscoring disparities in regional research visibility and output.
3) **Colombia and Costa Rica exhibit significant strengths in biomedical research capacities, while other countries face challenges**. According to the survey—as reported in the Results section (Figure 4)—these disparities highlight the uneven scientific progress across the region. Colombia, specializing in Immunology and Microbiology/Parasitology, leads in institutional frameworks, research teams, and government funding. Costa Rica, excelling in Genetics and Immunology/Cellular Biology, interdisciplinary collaboration, and foundational biomedical research, benefits from access to advanced infrastructure. Peru, with strengths in Microbiology (particularly Parasitology), struggles with funding and infrastructure. Panama, showing potential in Biotechnology and Bioinformatics, lacks support for interdisciplinary research collaboration. Guatemala, despite resource limitations and smaller research teams, demonstrates promise in private sector engagement and interdisciplinary research.
4) **Gaps in biomedical degree programs and early-career support hinder the development of a competitive research workforce**. As reported in survey results on training programs and early-career support, Costa Rica and Colombia demonstrate relative strengths at the undergraduate and master's levels, as well as in early-career researcher support—including access to training and professional development. Guatemala exhibits significant gaps, particularly at the doctoral and postdoctoral levels (Figure 5). Peru shows promise in PhD programs and research skills training but faces challenges in providing access to internships and mentoring. Panama's lack of undergraduate programs and limited master's offerings may hinder the development of a robust pipeline of researchers. These disparities point to the importance of investing in infrastructure and faculty development, alongside fostering regional collaboration to accelerate capacity-building.
5) **Persistent challenges hinder biomedical research progress**. The survey reveals major and interconnected obstacles to biomedical research success across the studied Latin American countries (Figure 6). Limited government funding and an overreliance on precarious external financing pose significant challenges. These findings, reflected in Figures 2, 6, point to widespread funding constraints that undermine long-term research planning. Limited domestic investment and reliance on external sources reduce institutional flexibility. Addressing this will require greater baseline funding and more effective coordination of international support. Inadequate infrastructure, particularly in Peru, Panama, and Guatemala, further restricts research progress. Shortages of qualified researchers, especially in Peru and Guatemala, represent a critical bottleneck. Additionally, political and economic instability—particularly in Guatemala and Colombia—along with limited international collaboration, further threaten sustained research capacity.

### Interpreting the research landscape

Differences in biomedical research capacities and performance across the studied countries are closely tied to socioeconomic conditions and R&D investments. While specific data on biomedical-related R&D spending as a percentage of GDP remains unavailable, medical sciences account for 10%−20% of total R&D expenditures in Latin America, according to ECLAC (2023). Each country's ability to sustain a robust biomedical research ecosystem depends on factors such as GDP per capita, R&D expenditures, workforce development, and innovation outputs.

Our data show that regional R&D leaders (Brazil, Mexico, and Argentina) exhibit a complex relationship between research investment and output (Tables 2, 3). While Brazil leads in R&D investment, researchers, and publications, Argentina demonstrates higher per-capita researcher efficiency, and Brazil leads in per-capita R&D expenditure. Mexico ranks second in most metrics, but Chile demonstrates higher per-capita R&D expenditure efficiency. Among the study countries, Colombia ranks fifth overall. However, despite lower H-indices, Costa Rica (ranked 12th) exhibits higher per-capita R&D expenditure efficiency than Mexico (ranked second), Chile (ranked fourth), and even Colombia (fifth). This suggests that factors beyond budget size, such as infrastructure, collaboration, and targeted funding, may influence research productivity. For example, Costa Rica's higher R&D investment and structured STI strategy provide a significant advantage. In contrast, Panama, despite having the highest per capita income among the studied countries, struggles with fragmented policies, limited funding, and extreme income inequality. These contrasting examples illustrate that while financial investment is important, effective strategies and supportive environments are equally crucial for maximizing research output.

Colombia's R&D investment, while modest, provides stability that, combined with a growing research workforce, enables its leading biomedical research output. Costa Rica's higher R&D investment and steady workforce expansion contribute to a more stable research environment. Conversely, Guatemala's minimal R&D investment and limited research workforce restrict long-term scientific growth.

The region's low R&D investment reflects global trends in developing regions (UNESCO, 2021), hindering the growth of competitive research ecosystems and increasing reliance on external funding. This funding gap is widening, with Latin America and the Caribbean spending nearly four times less on R&D (as a percentage of GDP) than developed nations and emerging economies. As a result, scientific output remains limited; in 2019, the region accounted for only 5.3% of global scientific publications (UNESCO, 2021). Even countries with stronger research capacities, such as Colombia and Costa Rica, depend on international collaborations, highlighting the crucial need for sustained domestic investment. Increased R&D investment correlates with greater innovation output, as demonstrated by countries such as South Korea and Israel (World Bank, 2022). Without substantial increases in domestic funding, Latin American research systems will struggle to achieve sustainable, long-term growth and compete globally.

Beyond formal degree programs, our survey confirms significant shortfall in resources for students and early-career researchers across the region. While Colombia, Costa Rica, and Peru offer more structured programs for graduate students and early-career researchers, Guatemala and Panama face critical gaps due to limited institutional resources and funding constraints. Hands-on training programs, such as internships and laboratory rotations, are particularly emphasized in Colombia and Costa Rica, reflecting institutional efforts to bridge the gap between academic training and practical research experience. Meanwhile, Peru's progress in PhD training is hindered by insufficient mentorship and professional development opportunities, which restricts career advancement for young researchers. These gaps highlight the link between investment and training capacity observed in the degree program analysis and emphasize the need for targeted support to professionalize scientific careers throughout the region. A similar pattern emerges when examining postdoctoral training opportunities. While interest in international postdoctoral programs is substantial, awareness and perceived accessibility remain key factors influencing participation rates. These findings further underscore the need for targeted outreach, enhanced language support, and stronger institutional mechanisms to encourage greater engagement in global research opportunities.

Research output, often measured by scientific publications, is strongly correlated with the size of the R&D workforce (Rosenbloom et al., 2015). Despite recent growth in scientists and research institutions, Latin America still lags significantly behind developed countries in research capacity (Ciocca and Delgado, 2017). Among the five countries in our study, Colombia's larger research teams facilitate interdisciplinarity. While Costa Rica has high-quality faculty, its teams are medium-sized. In Peru, Panama, and Guatemala, team research productivity and innovation are restricted by infrastructure shortages. Furthermore, the limited number of qualified researchers in Peru and Guatemala, coupled with weak professional incentives, further limits progress. Political and economic instability in Guatemala and Colombia exacerbates these challenges, hindering sustained long-term research investments.

National funding priorities influence institutional capacity and specialization by directing resources toward specific areas of study. Funding imbalances across US biomedical research fields likely stem from several factors, including funding agency leadership priorities and the perceived potential for major discoveries in certain fields (Ioannidis et al., 2022). Our survey revealed distinct specializations across the selected countries, which could potentially reflect funding availability at either the national or international level. Colombia's prominent fields were Immunology and Microbiology/Parasitology, complemented by strengths in Neurosciences. Peru's dominant specialization was Microbiology/Parasitology, reflecting a focus on infectious diseases, followed by Biochemistry, indicating an emphasis on molecular-level research. Costa Rica focused on Genetics and Immunology/Cellular Biology. Guatemala demonstrated a more diversified research landscape, with Genetics, Immunology, and Microbiology/Parasitology all represented. Panama emphasized technologically advanced fields such as Biotechnology and Bioinformatics/Computational Biology.

These findings align with existing research, including our targeted literature review on biomedical research capacities in Latin America, which highlights recurring regional strengths and challenges across various specializations.

In immunology, Colombia leads with both independent and collaborative research (Fabila-Castillo et al., 2021), while Peru is expanding through global partnerships. Costa Rica is developing its capacity for independent research, whereas Guatemala struggles with productivity challenges. In microbiology research, Nai's 2013–2014 study (2017) highlights Colombia's progress, contributing 4% of total Digital Object Identifiers (DOIs), indicating a growing research landscape. In contrast, Peru's lower output (< 1% of DOIs) reflects ongoing challenges in building a robust research environment. Regionally, Brazil (64%) and Argentina (23%) dominate, while Chile (5%) and Uruguay (2%) contribute smaller shares.

Colombia's virology research has grown substantially, with publications increasing between 2000 and 2013 (Ruiz-Saenz and Martinez-Gutierrez, 2015). These advancements reflect improved collaboration, productivity, and publication quality. Similarly, Colombia is emerging as a key player in Latin American neuroscience research (Forero et al., 2020), actively contributing to scientific advancements despite funding and infrastructure challenges. While Brazil and Mexico lead in publications and citations, Colombia continues to strengthen its presence in the field.

Latin American epidemiological research is uneven (Barreto et al., 2012). Among the five studied countries, Colombia, Peru, and Costa Rica show strengths; Panama and Guatemala face limitations. Costa Rica's bioinformatics has expanded through improved programs and infrastructure (Campos-Sánchez et al., 2021), despite challenges in building research mass. Furthermore, developmental biology is an emerging field in Latin America. Colombia leads with extensive studies in evolutionary developmental biology and regeneration, while Costa Rica utilizes its biodiversity for health-related and non-traditional model system research. Panama has also significantly advanced developmental biology through diverse research initiatives (García-Arrarás, 2021).

To assess biomedical research capacities, this study gathered insights from key actors in Latin America's research ecosystem. Interviews and surveys captured perspectives from researchers, professionals, and stakeholders, providing a deeper understanding beyond official reports. Their input highlights current conditions, aspirations, strengths, opportunities, and critical challenges. Persistent obstacles, including government underfunding, weak infrastructure, and limited workforce capacity, continue to hinder biomedical research development.

Addressing these disparities requires targeted investments in training, institutional support, and sustainable funding mechanisms to maximize opportunities, foster scientific innovation, and enhance Latin America's global research competitiveness.

### The role of international programs on regional research capacities: the case of the Pew program

Our study examines the role of the Pew Latin American Fellows Program in strengthening research capacities, using it as a case study of international programs. Given the limited postdoctoral training opportunities in many Latin American institutions, initiatives such as the Pew Program are critical for exposing researchers to high-impact scientific environments. The program provides 2 years of postdoctoral salary support in the U.S., networking opportunities, and seed funding for fellows returning to establish their own labs in Latin America, thereby contributing to regional research capacity.

Since its inception in 1991, the Pew Latin American Fellows Program has awarded 331 fellowships. Historically, between 1991 and 2023, the program has shown a strong concentration of applications and awards within Argentina, Brazil, Chile, and Mexico. These four countries collectively submitted approximately 86% of applications and received 78% of the awards. In contrast, other Latin American countries, including Colombia, Ecuador, Peru, Uruguay, and Venezuela, submitted approximately 14% of the applications and received 22% of the awards. This disparity reveals a significant imbalance in participation and highlights the need to expand opportunities across the region, especially in countries with limited research resources.

These data also show that, despite submitting fewer applications, applicants from these countries, which also include Costa Rica, Guatemala, and Panama, have demonstrated strong competitiveness, achieving the highest success rates among all groups. This suggests that low application numbers, rather than applicant quality, are the primary limitation to achieving more balanced regional representation.

Recent trends indicate a shift. There has been an increase in applications from these other countries, coupled with a decrease in applications from Argentina and Chile. These trends offer valuable insights into the ongoing disparities in application numbers and success rates across Latin America, underscoring the persistent dominance of a few countries in both the applicant pool and fellowship distribution. Expanding outreach efforts and strengthening institutional mechanisms to guide and support potential applicants in these less participating additional countries would be essential to achieving a more balanced participation in the program.

### Opportunities and obstacles

This study reveals key opportunities and obstacles related to the Pew Program's role in supporting biomedical research capacities in the selected countries.

First, the Pew Program's low visibility represents a significant missed opportunity for talented researchers in the region, particularly in Guatemala, Panama, and Peru, where the pool of qualified applicants is already limited. Moreover, access is hindered by compounding obstacles, limiting the program's impact on regional research capacity.

Second, barriers for participation in the Pew Program include financial constraints, language proficiency, and limited institutional support. Specifically, English proficiency is a challenge in Colombia and Peru, while Guatemala struggles with a limited PhD pool and doctoral programs. Panama lacks postdoctoral training opportunities. Inadequate institutional support and mentorship exacerbate these issues across all countries.

Third, despite regional biomedical strengths—Costa Rica in genetics, Colombia in infectious diseases, Panama in biotechnology, and Peru in microbiology—structural limitations, including funding and infrastructure challenges, hinder Pew Program participation. To address this, diversified funding, improved infrastructure, and stronger international collaboration are essential. These strengths in genomics, immunology, and biopharmaceuticals present a strategic opportunity to align with emerging bioeconomy initiatives in Latin America. By leveraging established capabilities in drug discovery, vaccine development, and biomedical innovation, the region can drive sustainable economic growth while addressing regional and global health challenges. Effective integration into bioeconomy policy strategies is critical for achieving this synergy, as highlighted by Rodrigues et al. (2019).

### Enhancing program impact and reach

These findings underscore both the vital importance of international fellowships and the systemic barriers limiting their impact, particularly in countries with the greatest need. Without addressing these fundamental issues, researchers will continue to face limited access to cutting-edge knowledge and international collaborations, ultimately affecting the region's research capacity and scientific development.

Several key areas emerge as critical for addressing these challenges: increasing program visibility through targeted outreach, supporting language development initiatives, strengthening institutional support networks, and improving research infrastructure. These areas require particular attention in countries such as Guatemala and Peru, where postdoctoral training remains undervalued.

To enhance program participation, universities and research institutions in Latin America need to develop formal structures supporting early-career researchers. Specific measures should include establishing mentorship programs, providing English-language training, and funding preparatory courses. Stronger collaboration between Pew alumni and local institutions would enhance visibility while providing practical guidance to prospective applicants, especially in regions where awareness is low.

The program's long-term success depends on ensuring that postdoctoral training translates into tangible career benefits. By implementing these targeted interventions and strengthening institutional support mechanisms, the program can maximize its contributions to the region's research ecosystem while broadening access for qualified candidates facing linguistic or administrative barriers.

### Expanding international research support

Beyond the Pew Program, our findings highlight the broader importance of international fellowships in reducing research gaps and enhancing scientific productivity in Latin America. Research mobility programs are well-documented catalysts for fostering collaboration, improving research quality, and driving innovation in low-resource settings. Countries that have successfully expanded their research output—such as Brazil and Argentina—have done so in part through increased participation in international collaborations and fellowship programs.

However, Latin American countries face distinct challenges that international programs need to address to maximize their effectiveness. Strengthening local research infrastructure is critical so that researchers returning from international fellowships have adequate facilities to continue high-level research. Enhancing career pathways for returning researchers can prevent “brain drain” and ensure postdoctoral training leads to concrete professional opportunities.

Our findings emphasize the valuable role of international fellowship programs in fostering biomedical research capacity in Latin America, while also highlighting significant structural barriers that limit participation. The Pew Latin American Fellows Program represents a critical opportunity for regional researchers, but its impact remains constrained by low awareness, financial and linguistic obstacles, and weak institutional support.

### Implications for capacity building and policy recommendations

Our study reveals persistent gaps and opportunities for strengthening biomedical research capacity across Latin America. Specific challenges, such as the institutional deficits in research training and infrastructure highlighted in the Results (Figures 3, 4), reinforce the need for targeted investment in human capital and facilities, as outlined below. These observations underscore the need for a multifaceted approach that integrates infrastructure development, human capital investment, and enhanced collaboration to build a more resilient and internationally competitive biomedical research ecosystem. Based on these findings, we propose the following key areas for improvement and policy recommendations:

1. **National R&D Investment:** Governments must prioritize increasing public funding for biomedical research, particularly in countries with the lowest R&D expenditure as a percentage of GDP, such as Guatemala, Panama, and Peru. While international organizations such as UNESCO recommend 1%−3% of GDP for R&D (UNESCO, 2016), a significant gap remains between these guidelines and implementation, particularly evident in smaller Central American countries with recently established STI policies (Padilla-Pérez and Gaudin, 2014). This investment must be coupled with the establishment of sustainable funding mechanisms to provide long-term support for research initiatives.
2. **Policy Frameworks:** Governments should implement comprehensive national roadmaps that align policies with global best practices and decentralize research activities beyond major urban centers to promote more inclusive scientific development. Since the 2000s, Science, Technology, and Innovation (STI) policies in countries such as Argentina and Brazil have prioritized health, biomedicine, nanotechnology, and biotechnology, aligning research with national development goals (Sandoval-Romero et al., 2018). Targeted policies could further strengthen these fields in the selected countries. These policies should specifically address the need for enhanced training programs and strategic international partnerships.
3. **Building Institutional Capacity:** Modernizing laboratories and research centers through investment programs and public-private partnerships is essential for enhancing biomedical research productivity. This includes establishing centralized grant-writing and research administration offices to improve efficiency in securing and managing funds, streamlining administrative processes. Strengthening collaborations between universities and hospitals can facilitate the integration of biomedical research with clinical applications, increasing the societal impact of scientific discoveries. Prioritizing research infrastructure modernization is crucial to ensure laboratories and facilities meet international standards.
4. **Developing Human Capital:** Sustainable training and capacity building for Latin American biomedical research depends on consistent institutional support and well-defined career pathways. Stable funding and project availability are required for hands-on research experience for students, postdoctoral fellows, and early-career professionals. This foundation is particularly critical in Peru, Guatemala, and Panama, where there is a severe shortage of PhD researchers and limited postdoctoral opportunities. International postdoctoral programs, such as Pew, serve as vital bridges between training and independent research careers, helping to expand networks and strengthen regional expertise. Expanding these opportunities through targeted scholarships, research incentives, and institutional support can help address current gaps in graduate and postdoctoral training. Long-term sustainability requires comprehensive support mechanisms. These include enhancing competitive salaries, creating tenure-track positions, and ensuring stable funding to retain skilled researchers and reduce brain drain. Additionally, integrating English training and professional communication skills into graduate programs can increase participation in international scientific networks, increasing the global visibility and impact of Latin American researchers. Human capital development programs need expansion to build a robust pipeline of skilled researchers.
5. **Regional and International Collaboration:** To address STI asymmetries in Latin America and the Caribbean, greater regional integration through international and regional cooperation is crucial. Colombia and Costa Rica, with more developed biomedical research ecosystems, should lead in fostering regional partnerships. Strengthening research networks, especially in underfunded fields, can facilitate knowledge sharing. Joint funding initiatives with international organizations and improved fellowship outreach can broaden participation in global research. Argentina, Brazil, and Mexico (particularly Brazil, with 1.2% of GDP in 2020) account for the majority of regional R&D spending (ECLAC, 2024) and are the most productive (Zacca-González et al., 2018; León-de la O et al., 2018), making their involvement in these collaborations essential. Such initiatives should include regional graduate training programs, postdoctoral fellowships, and collaborative projects. These collaborations are vital for securing stable financial resources and fostering strategic international partnerships.

The key challenges hindering biomedical research capacity in Latin America, along with their corresponding policy areas and actionable strategies, are summarized in Table 5. This structured framework highlights targeted interventions needed to strengthen research funding, infrastructure, human capital, and international collaboration. By implementing these strategies, Latin America can build a more robust and sustainable biomedical research ecosystem, better equipped to address regional and global health challenges while contributing more significantly to scientific advancement.

**Table 5:** Key challenges, policy areas, and actionable strategies.

**Key challenges**	**Corresponding policy area(s)**	**Actionable strategies**
1. Inadequate R&D investment and dependence on external funding	- Increasing and diversifying research funding - Implementing supportive policies for sustainable research growth	- Advocate for policies that allocate a higher percentage of GDP to R&D - Develop public-private partnerships to diversify funding sources - Establish national research endowments to provide sustainable funding - Improve grant-writing support to increase success rates in international funding applications
2. Fragmented STI systems	- Implementing supportive policies for sustainable research growth	- Develop national and regional STI strategies with clear long-term objectives - Strengthen coordination between government agencies, universities, and research institutions - Establish coordinated national research agencies to improve funding distribution and policy coherence
3. Limited research infrastructure	- Enhancing research infrastructure and innovation ecosystems - Increasing and diversifying research funding	- Invest in modernizing laboratories and research facilities - Create regional shared research centers to maximize infrastructure use - Promote public-private initiatives for funding advanced equipment acquisition
4. Insufficient critical mass of researchers	- Strengthening institutional and human capital development	- Expand PhD and postdoctoral training programs through targeted scholarships - Support faculty development programs to retain researchers - Increase funding for early-career researchers to establish independent research projects
5. Insufficient training opportunities and researcher development	- Strengthening institutional and human capital development	- Strengthen mentorship and professional development programs - Expand hands-on training initiatives, including laboratory rotations and internships - Enhance interdisciplinary and translational research training
6. Career development barriers and brain drain	- Strengthening institutional and human capital development - Implementing supportive policies for sustainable research growth	- Develop reintegration programs to support returning researchers - Provide stable employment and clear career progression pathways to retain talent - Strengthen career pathways by linking research to industry and policy sectors
7. Socioeconomic inequalities and limited public support	- Strengthening institutional and human capital development	- Expand STEM education initiatives in underprivileged areas - Foster inclusive research policies - Increase public engagement with science
8. Political and economic instability	- Implementing supportive policies for sustainable research growth	- Advocate for stable, long-term research funding commitments independent of political cycles - Strengthen national research institutions to ensure continuity of programs - Develop contingency plans for research continuity during economic downturns
9. Health crises and global health challenges	- Enhancing research infrastructure and innovation ecosystems - Implementing supportive policies for sustainable research growth	- Prioritize local health research programs, such as infectious diseases - Strengthen regional collaboration for rapid response to health crises - Develop funding mechanisms to sustain biomedical research beyond immediate crises
10. Barriers to international collaboration	- Facilitating international collaboration and research networks	- Simplify regulatory frameworks for research collaborations - Provide financial and logistical support for international exchanges - Strengthen language training programs to improve researcher participation in global initiatives

### Learning from global experiences and international funding models

The challenges identified in our study are not unique to Latin America. Countries across Africa and Asia—many of which are non-English-speaking—have encountered similar barriers related to language, internationalization, and research capacity-building. Their experiences offer valuable lessons that could inform strategies within the Latin American context.

For instance, international teacher exchange programs in Morocco (Oubit and El Farahi, 2024) and extensive international partnerships in sub-Saharan Africa (Nyirenda et al., 2021) have been pivotal in promoting language acquisition, cultural understanding, leadership development, and robust research capacity. Crucially, successful African initiatives emphasize that capacity-building extends beyond mere financial support, prioritizing shared goals, mentorship, local ownership, and effective capacity transfer (Dean et al., 2017; Whitworth et al., 2010). China's experience further underscores the importance of institutional support for international scientific engagement, with reforms since the 1990s emphasizing English proficiency, publishing in high-impact international journals, and strategic partnerships significantly boosting its global scientific presence (Zhou and Leydesdorff, 2006; Shu et al., 2022; Jin and Rousseau, 2004). As Coelho et al. (2019) emphasize, prioritizing scientific English within graduate training is essential for non-native speakers to participate meaningfully in global research networks. These global examples collectively highlight the importance of integrating language training, international communication skills, and robust institutional support mechanisms into comprehensive research capacity-building strategies in Latin America.

Moreover, the global research funding landscape offers diverse strategies that can inform and enrich Latin American capacity-building efforts. Comparative examples from other regions demonstrate that aligning financial support with mentorship, institutional development, and infrastructure investment is essential for long-term success. As Lah (2017) emphasizes, effective research capacity-building hinges on strategic, sustained investments that integrate funding with mentorship, institutional development, and long-term partnerships. The Wellcome Trust, for instance, provides international fellowships and grants in Africa and Asia that effectively combine funding with mentorship and institutional strengthening (Whitworth et al., 2010), fostering both professional development and institutional resilience. Similarly, the European Union's Horizon programs have advanced research ecosystems in non-EU countries through structured partnerships and targeted capacity-building (Schuch et al., 2012). The deepening EU-CELAC STI cooperation since 2010 provides a strong example of bi-regional collaboration and shared investment in research capacity within Latin America itself (Sánchez, 2018).

Within Latin America, agencies such as the Inter-American Development Bank, the Pan American Health Organization (PAHO), Germany's DAAD, and Spain's AECID actively fund research but could be more effectively leveraged to support researcher training, career development, and institutional capacity. These organizations offer a range of funding opportunities and collaborative programs vital for mitigating brain drain and building sustainable national research ecosystems. Incentives such as return-to-country programs and structured academic pathways, as emphasized by Kupfer et al. (2004), are crucial for retaining talent and fostering a robust pipeline of skilled researchers.

Together, these international experiences underscore the value of diversified, long-term engagement with global funders. By developing stronger institutional frameworks and sustained partnerships informed by these global best practices, Latin American countries can significantly improve research output, retain skilled researchers, and reduce dependency on fragmented or short-term funding sources.

### Limitations and future research

This study offers valuable insights into biomedical research capacities in selected Latin American countries, but it has limitations that should be considered. The use of non-probability sampling limits the generalizability of survey results. Self-reported data may introduce biases. Additionally, the limited number of expert interviews constrains the depth of qualitative insights.

Future research should address these limitations by employing more representative sampling methods, conducting longitudinal studies, and exploring in-depth case studies. A more detailed examination of each country's specific challenges in accessing Pew Program postdoctoral fellowships would be valuable, and further analysis of individual country experiences is generally needed to fully understand the role of international funding agencies in biomedical research capacity building. This is particularly important given that insufficient research capacity, especially the imbalance in research production and utilization between high-income and low-/middle-income countries, hinders evidence-based health practices and policy, exacerbating health inequalities (Tulloch-Reid et al., 2018). Because most Latin American nations fall into the lower-middle or upper-middle income brackets, further research, beyond the well-studied Brazil, Argentina, Mexico, and Chile, is essential to address this gap and improve regional health outcomes. Expanding research on the role of international programs such as the Pew Latin American Fellows Program can provide further insights into their long-term impact on research capacity building in the region.

## Conclusion

This assessment of biomedical research capacities in Colombia, Peru, Guatemala, Costa Rica, and Panama reveals significant disparities in R&D investment, research infrastructure, workforce development, and institutional support. While Colombia and Costa Rica exhibit stronger research ecosystems, Guatemala, Panama, and Peru face limited national funding, dependence on external grants, and weak postdoctoral training opportunities. These limitations are further exacerbated by socioeconomic inequalities, fragmented science policies, and regulatory inefficiencies, restricting regional research growth.

Despite these challenges, Latin America possesses unique biological resources and growing specialization in key biomedical fields—including genetics, immunology, biotechnology, microbiology, and bioinformatics—that can serve as engines for innovation. However, realizing this potential requires targeted interventions. Increasing R&D investment, expanding structured PhD and postdoctoral programs, modernizing laboratory infrastructure, and creating sustainable national funding mechanisms are critical steps to strengthen biomedical research and reduce dependency on external grants.

Expanding regional and international collaborations will be essential in addressing shared barriers, promoting knowledge transfer, and improving research resilience. Programs such as the Pew Latin American Fellows Program illustrate the transformative role of international initiatives, particularly in postdoctoral training and talent retention. Strengthening regional research consortia, public-private partnerships, and coordinated policy frameworks will further enhance Latin America's ability to contribute to global biomedical advancements.

Biomedical research serves a dual purpose: advancing scientific knowledge while addressing urgent public health needs. Aligning R&D investments with regional priorities can stimulate innovation, reduce inequalities, and drive economic growth.

This study provides evidence-based insights for governments, funding agencies, and academic institutions to implement policies that bridge existing gaps in science policy, research training, and capacity building. By fostering a more inclusive and resilient research ecosystem, Latin America can position itself as a key player in global biomedical science and health innovation.

## Data Availability

The original contributions presented in the study are included in the article/supplementary material, further inquiries can be directed to the corresponding author.
